# Intra-Individual Variability in Sagittal Plane Kinematics During Indoor Cycling Time Trial †

**DOI:** 10.3390/sports13040110

**Published:** 2025-04-07

**Authors:** Chris Whittle, Simon A. Jobson, Neal Smith

**Affiliations:** 1 Department of Sport, Exercise and Health, University of Winchester, Sparkford Road, Winchester SO22 4NR, UK; simon.jobson@winchester.ac.uk; 2 Department of Sport & Exercise Sciences, University of Chichester, College Lane, Chichester PO19 6PE, UK; n.smith@chi.ac.uk

**Keywords:** movement variability, continuous relative phase

## Abstract

Intra-individual movement variability has historically been discounted as evidence of poor motor control. However, evidence now suggests that it may play a functional role in skill performance and so this study aimed to establish whether this is the case during a simulated indoor cycling time trial. Ten trained cyclists (Age = 31.90 ± 10.30 years, Height = 1.80 ± 0.10 years, Mass = 72.10 ± 9.40 kg) participated in a 10-mile (16 km) time trial while sagittal plane kinematics were captured using 3D motion capture technology. The results showed significant differences (*p* < 0.05) between knee–ankle and hip–knee coordination variability across pedal phases, with the knee–ankle coupling exhibiting more variability. Notably, faster cyclists demonstrated lower variability, particularly in the knee–ankle coupling, compared to slower cyclists. While no consistent relationship was found between movement variability and time trial performance across all participants, the results suggest that there may be a link between the level of intra-individual movement variability displayed by a cyclist and the time in which they were able to complete a 10-mile simulated time trial task in laboratory conditions.

## 1. Introduction

Within cycling, the most common approach to motion analysis is to focus on individual lower extremity joints [1,2], specifically in the sagittal plane, due to the lack of motion observed in the frontal or transverse planes [3]. This approach can provide valuable information about joint motion, but it does not consider that the motion of one segment subsequently influences the motion of an adjacent segment, and therefore does not effectively capture the complexity of the coordinated motion of body components [4]. The existence of this coupling relationship between segments has been well established in gait-based kinematic investigations [5,6,7] but has only more recently been recognised as crucial in the analysis of cycling [8].

Within cycling, there is also limited investigation into intra-individual movement variability as it has historically been assumed that this is either detrimental to normal function or purely evidence of random noise within the neuromuscular or measurement system [9,10,11,12]. In contrast, however, there is growing evidence that intra-individual movement variability may perform a functional role in task performance [13], especially when the task requires adaptability of complex motor patterns within dynamic performance environments [14,15] and may enable greater adjustment for both intrinsic and extrinsic factors, which may influence an athlete’s performance. Evidence of such adaptations in skilled performers has been established in a range of sports, with authors concluding that variability may play a functional role in producing a more consistent sporting outcome, despite the altering demands placed on the performer [13]. It seems, therefore, that intra-individual movement variability should be viewed as a form of “essential noise” [16].

There are a variety of methods available to quantify movement variability [17], all of which are cognisant of the idea that joint movements do not happen in isolation due to the interconnected nature of the structures within the human body. This is especially true when one end of the kinetic chain is attached to a pedal and, as such, it has been suggested that the consideration of the coupling relationship between segments may therefore be especially crucial in the analysis of motion within cycling [8]. 

Methods such as Discrete Relative Phase illustrate the relative timing of key events in a movement cycle, allowing for a measurement of latency between, for example, the maximum flexion of one joint compared to that of another. The disadvantage of these methods, however, is that they only take a measurement of this co-ordination once per movement cycle [18]. In the case of this particular investigation, this would be the equivalent of only measuring the relative position of two joints once per pedal revolution. 

Continuous relative phase analysis overcomes this issue as it offers the ability to evaluate movement coordination, and therefore variability, over a complete movement cycle [8]. This is achieved by replacing the angle plots of a relative motion approach with phase plots, which can then be used to calculate the four-quadrant arctangent phase angle of the joints of interest. This allows for the calculation of the relative phase between two segments at every point in the trajectory [19]. Once the phase angles are calculated and the time history is normalised to a fixed number of data points, the continuous relative phase is found by simply subtracting the phase angle of one joint from that of the other at each point in time over the entire movement [8,19,20].

Continuous relative phase was deemed most appropriate for this investigation due to the continuous, multijoint nature of the cycling task [21], as well as it being more sensitive to changes in coordination [22]. In addition, calculations of continuous relative phase provide a measure which is sensitive to the effects of fatigue, learning or other independent variables [23], which is important when analysing human movement from a dynamical systems perspective.

One article [24] which does investigate intra-individual movement variability within cycling seems to initially support the traditional motor learning theories in viewing variability as indicative of an unskilled performance. However, with the study design and analysis methods of the previous article able to be improved, this investigation will investigate if lower extremity intra-individual movement variability alters in cyclists of differing performance levels, and if this plays a functional role in the completion of a simulated indoor time trial event.

## 2. Materials and Methods

### 2.1. Participant Information

Ten trained cyclists volunteered to take part in this study (see Table 1). Participants all held a current British Cycling Race License (Category 1 n = 1, Category 2 n = 2, Category 3 n = 2, Category 4 n = 5) and mean training load was self-reported as 10.85 ± 4.21 h or 156.00 ± 48.35 miles per week. Participants maintained their normal diet and daily activity patterns throughout the testing period and informed consent was obtained from all participants involved in this study. Local ethics approval was provided by the University of Winchester.

### 2.2. Testing Procedure and Instrumentation

Initial testing consisted of a graded exercise test (GXT) to establish V^·^O_2_ max values for each participant. This was to ensure physiological similarities across the sample so as to remove the confounding variable of physiological differences when assessing movement variability. An electromagnetically braked cycle ergometer (SRM GmbH, Jülich, Germany) was used to conduct a continuous incremental cycling GXT where workload was increased by 5 W per 15 s. The initial workload was adjusted according to the participants’ self-reported estimate of maximal power output so that the total duration of the GXT was between 8 and 10 min. Criteria for termination of the maximal GXT was primarily based on volitional exhaustion.

Throughout the GXT, online respiratory gas analysis was performed using a breath-by-breath automatic gas exchange system (MetaLyzer 3B, Cortex Biophysik GmbH, Leipzig, Germany) following volume and gas calibration. Heart rate (HR) was monitored using a wireless chest strap telemetry system (Polar Electro T31, Kempele, Finland), as well as ratings of perceived exertion every minute using the Borg 6-20 RPE scale. Maximal oxygen consumption was recorded as the highest average oxygen consumption over a 60 s period.

Participants then visited the laboratory on 3 occasions, separated by a minimum of 48 h to allow full recovery between trials. During each testing session, reflective markers (Qualisys, Gothenburg, Sweden) were attached to the greater trochanter, lateral epicondyle of the femur, lateral malleolus and 5th metatarsal on both sides of the participant’s body, as well as a reflective marker on each pedal. Participants undertook a self-directed warm-up followed by a simulated 10-mile (16 km) time trial and self-directed cool down. Time trials were conducted from a standing start and participants were given free choice of gearing and cadence throughout.

All time trials were conducted in an air-conditioned laboratory using a Wattbike Pro cycle ergometer (Wattbike Ltd., Nottingham, UK). Participants used their own cycling shoes. The ergometer was set to, as closely as possible, replicate the dimensions of each participant’s own bicycle, and participants were given access to any data they would normally ride with to monitor their cycling effort (e.g., cadence, heart rate and power output).

A 12-camera motion capture system (Qualisys Oqus 300+, Gothenburg, Sweden) sampling at 500 Hz recorded three-dimensional kinematic data at the hip, knee and ankle throughout each trial via Qualisys Track Manager (Version 2019.2). Time trial completion time was retrieved from the Wattbike using Wattbike Expert software version 2.60.20 (Wattbike Ltd., Nottingham, UK).

### 2.3. Data Analysis

One time trial was selected per participant for analysis. This was the last performance to allow the first two trials to act as familiarisation sessions, unless, due to technical errors with marker adhesion, there was insufficient kinematic data to make this feasible. In this case, the most complete recording was selected.

Sagittal plane joint angle and joint angular velocities at the hip, knee and ankle were recorded for 10 complete pedal revolutions at 5 min intervals throughout the time trial. One revolution was identified as the time between the pedal reaching the top dead centre (0°) on two consecutive occasions. This was defined as the point where the pedal marker reached its maximal value in the vertical axis of the global co-ordinate system. Joint angle and angular velocity were then interpolated to 100 data points using a cubic spline technique.

The interpolated data was then used to calculate the continuous relative phase (CRP) to provide intra-limb couplings at: (i) knee flexion/extension–ankle plantarflexion/dorsiflexion (KA) and (ii) hip flexion/extension–knee flexion/extension (HK).

CRP was defined as the difference between the normalised phase angles of the coupling throughout the revolution, measured in degrees (°). CRP was reported on a linear scale of 0–180°, with 0° corresponding to a perfectly in-phase coupling, meaning that the phase angles for the two motions are identical, and 180° representing a perfectly anti-phase coupling.

#### 2.3.1. CRPv Testing

Replicating previous analysis methods [24], initial testing involved the calculation of continuous relative phase variability (CRPv), which was defined as the standard deviation at each data point across the 10 revolutions for each participant. This process was repeated for data sampled at 5 min, 10 min, 15 min and 20 min throughout the time trial effort. 

**Figure 1:**
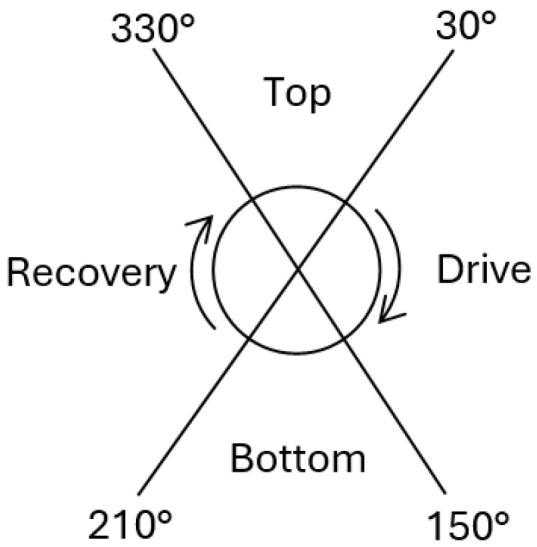
Four-phase division of a pedal revolution.

Each revolution was subsequently divided into four phases to produce separate top, drive, bottom and recovery phases [25], as shown in Figure 1. Mean CRPv values per phase were calculated for each. 

#### 2.3.2. Whole-Group CRPv Testing

Initially, analysis was conducted using the whole participant group to correlate each participant’s mean CRPv per pedal phase against the time taken to successfully complete the time trial (Time_TT_). This was conducted using Pearson’s product moment correlation co-efficient and repeated for each coupling (hip–knee and knee–ankle) of each leg (left and right) at each time point (5 min, 10 min, 15 min and 20 min). 

Subsequently, a two-way analysis of variance (ANOVA) was conducted to test for differences between time points (5 min, 10 min, 15 min and 20 min and couplings) (hip–knee and knee–ankle), as well as the interactions between these factors. This was repeated for each phase of the revolution (top, drive, bottom and recovery) to assess whether the amount of movement variability displayed by the participants varied throughout the time trial. 

#### 2.3.3. Split Group CRPv Comparisons

Following initial testing, the group was split into “faster” and “slower” groups at the point of the largest difference in Time_TT_ (between the 5th and 6th ranked riders). This gave an equal split of participants between groups (*n* = 5 in each). The statistical procedures outlined for whole-group testing were then repeated considering the faster and slower groups separately.

In addition, a series of one-way independent samples ANOVAs were conducted to investigate differences between faster and slower groups in terms of CRPv values in each pedal phase (top, drive, bottom and recovery) and over time (5 min, 10 min, 15 min, 20 min). 

#### 2.3.4. CV% Testing

To offer an additional measurement of variability, the coefficient of variation (CV%) of CRP values was calculated using the following formula:

Co-efficient of variation = (standard deviation of CRP/mean of CRP) × 100

This produced a percentage value (CV%) which represents the amount of variance each participant displayed in their joint couplings between the measurement time points (5 min, 10 min, 15 min and 20 min) throughout the simulated time trial. This additional calculation was designed to overcome the influence of the finite magnitude of a value on variability [26] and negate the tendency of standard deviation to unavoidably increase as the range of the measure increases. CV% is a unitless value and is divorced from any scale of measurement [27], and is therefore suggested as a clearer comparison of the true variance displayed.

A Pearson’s product moment correlation coefficient was calculated to test for the relationship between CV% and the time taken to complete the simulated 10-mile time trial (Time_TT_) for all riders.

This process was repeated using the same pedal revolution divisions described above and, as before, initial testing was conducted using the whole participant group to correlate each participant’s coefficient of variation in continuous relative phase values (CV%) against the time taken to successfully complete the time trial (Time_TT_). This was conducted using Pearson’s product moment correlation co-efficient and was repeated for both hip–knee and knee–ankle joint couplings. This was specifically designed to ascertain whether a relationship existed between the amount of variation a cyclist showed between measurement points and the time taken for them to complete the time trial.

All statistical testing was performed using IBM SPSS statistics version 24 (IMB Corporation, New York, NY, USA), with a significance level set at *p* < 0.05.

## 3. Results

Mean and standard deviation of CRPv values for the whole group can be seen in Table 2. The same data for the faster and slower groups are displayed in Table 3 and Table 4, respectively.

### 3.1. Relationship Testing

Across all testing for relationships between CRPv and Time_TT_ for the whole-group data, only the hip–knee coupling at fifteen minutes showed a statistically significant (*p* < 0.05) correlation. These results were r = −0.777, *p* = 0.014 for the top phase and r = −0.666, *p* = 0.050 for the drive phase, showing a statistically significant large negative correlation between CRPv and Time_TT_ at these points. All other correlations were not statistically significant.

Once the participants were split into faster and slower groups, the correlation of hip–knee coupling at 15 min with Time_TT_ remained statistically significant in the top phase of the revolution for the faster group (r = −0.975, *p* = 0.025) but was no longer significant for the slower group. The correlation of hip–knee coupling at 15 min for the drive phase was no longer statistically significant for either group.

In addition, the slower group showed statistically significant correlations between Time_TT_ and the following couplings: knee–ankle at 5 min in the top phase (r = −0.966, *p* = 0.008); knee–ankle at 5 min in the bottom phase (r = 0.922, *p* = 0.026); knee–ankle at 15 min in the top phase (r = −0.950, *p* = 0.050); knee–ankle at 20 min in the recovery phase (r = 0.988, *p* = 0.042). All other correlations were not statistically significant for both the faster and slower groups.

### 3.2. Difference Testing

For the whole-group testing, there was a significant difference (*p* < 0.005) between hip–knee and knee–ankle couplings during all pedal revolution phases, with the knee–ankle coupling showing consistently higher levels of CRPv across all time points than the hip–knee coupling.

Once participants were split into faster and slower groups, this remained the case for the slower group, while only the drive phase showed a significant difference (*p* = 0.013) between couplings for the faster group.

When comparing CRPv levels between the two groups, there was a statistically significant difference in the knee–ankle coupling at 10 min during the top phase (*p* = 0.024, faster group = 18.50° ± 7.54, slower group = 32.66° ± 7.23) and again at 20 min (*p* = 0.015, faster group = 24.15° ± 0.41, slower group = 37.84° ± 5.05). There were no other statistically significant differences between the groups.

There were no significant differences (*p* > 0.05) in CRPv over the course of the time trial when comparing between time points. This was the case for whole-group, faster-group and slower-group data.

### 3.3. CV% Testing

As seen in Table 5, all observed correlations were not statistically significant at an alpha level of *p* < 0.05. All relationships were negative except for the hip–knee joint coupling during the drive and bottom phases and the knee–ankle coupling during the drive phase when the revolution was split into four phases.

## 4. Discussion

The aim of the current study was to ascertain whether lower extremity intra-individual movement variability varies in cyclists of differing experience and if this plays a functional role in the completion of a simulated indoor time trial event.

### 4.1. Relationship Testing

**Figure 2:**
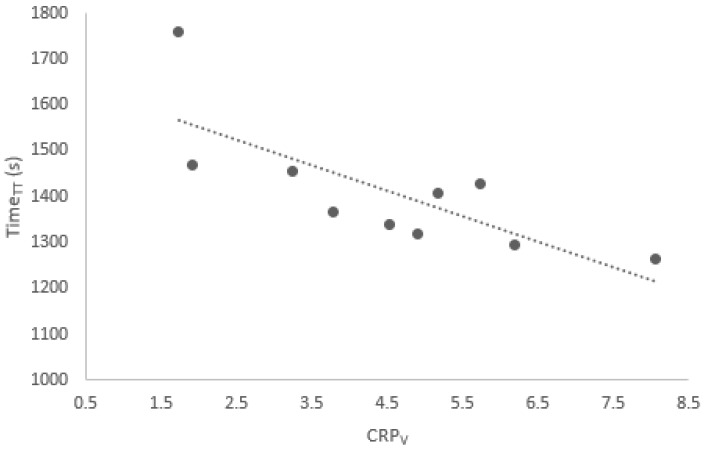
Correlation between Time_TT_ and CRP_V_ for hip–knee coupling at 15 min, for all participants during the “top” phase (r = −0.777).

**Figure 3:**
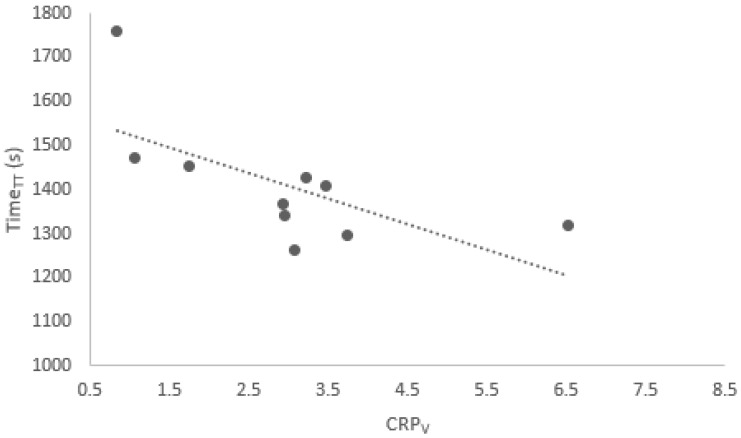
Correlation between Time_TT_ and CRP_V_ for hip–knee coupling at 15 min, for all participants during the “Drive” phase (r = −0.666).

The general lack of statistically significant correlations between CRPv couplings and Time_TT_ shows that there is little to no relationship between the level of intra-individual movement variability employed by participants and the performance outcome. The two significant correlations which were found for the whole group, however, were both negative (r = −0.777, *p* = 0.014 and r = −0.666, *p* = 0.050), suggesting that a greater level of movement variability may be linked to a faster time for the time trial event (see Figure 2 and Figure 3). This is in direct contradiction of previous studies [24], which concluded that movement variability is not beneficial to cycling performance. Instead, they suggested that out-of-phase motion reflects a less stable coordinative state [28], and that this may be indicative of the reduced effective force application [29].

Once the participants were split into faster and slower groups, the faster group only showed one statistically significant correlation between CRPv couplings and Time_TT_ (r = −0.975, *p* = 0.025), with the slower group showing four statistically significant correlations, none of which were present in the whole-group analysis. Of these four, two showed a positive relationship (right leg knee–ankle at 5 min in the bottom phase and left leg knee–ankle at 20 min in the recovery phase) and two showed a negative relationship (right leg knee–ankle at 5 min in the top phase and left leg knee–ankle at 15 min in the top phase).

Given the lack of consistency in terms of the direction of the relationship and the coupling, leg or time point in which the statistically significant correlations occur, it is difficult to reliably infer whether there is any functional role of intra-individual movement variability from this data.

### 4.2. Comparing Between Couplings

For the whole-group testing, there was a significant difference (*p* < 0.005) between hip–knee and knee–ankle couplings during all pedal revolution phases, with the knee–ankle coupling showing consistently higher levels of CRPv across all time points than the hip–knee coupling. This was expected as it has long been established that maximum knee and hip extension occur simultaneously at approximately 180° of the pedal revolution [30,31], whereas peak ankle dorsiflexion occurs around 90° and peak plantarflexion at approximately 285° [32].

Interestingly, once the participants were split into faster and slower groups, the significant difference in CRPv between hip–knee and knee–ankle couplings remained for the slower group, while only the drive phase showed a significant difference (*p* = 0.013) between couplings for the faster group. This could potentially be explained if the faster group were performing more of an “ankling” motion.

Ankling is a technique that involves pushing the pedal across the top of the pedalling cycle (0°) with the foot in the dorsi-flexed position and pulling across the 180° point of the cycle with the foot plantar flexed [33]. This has been demonstrated to occur more in elite cyclists than novices [8], and would potentially produce a more in-phase motion in terms of a knee–ankle coupling, explaining the lack of significant differences between couplings in the faster group’s data.

### 4.3. Comparing Between Time Points

Following previous work [31,34,35], which reported changes in the kinetics or kinematics of the cycling action as a result of fatigue, it was initially thought that the level of CRPv shown by participants may change over time. It was thought that participants may employ a variable coordination pattern in order to alter the muscle fibres recruited during each successive pedal revolution and therefore afford fibres momentary opportunities for recovery and preserve global task performance. This would represent an attempt to overcome decreased contractile properties of muscles during fatigue, clearly suggesting that a new movement pattern was employed in reaction to a changing set of task constraints.

Given that participants reported a mean RPE of 18.6 ± 1.7 (a rating of extremely hard to maximal exertion) upon completion of the time trial, it is fair to assume that a level of fatigue was present, but this did not manifest in any significantly different levels of CRPv across time points. This was true regardless of whether whole-, faster- or slower-group data was investigated. This is perhaps not overly surprising given the suggestion that the effect of fatigue on movement variability cannot be generalised across athletes [36], and further investigation is required to ascertain the muscular recruitment strategies employed throughout the time trial.

### 4.4. Comparing Between Groups

The final comparison of CRPv levels between the faster and slower groups showed there was a statistically significant difference in the right leg knee–ankle coupling at 10 min during the top phase (*p* = 0.024, faster group = 18.50° ± 7.54, slower group = 32.66° ± 7.23) and again at 20min (*p* = 0.015, faster group = 24.15° ± 0.41, slower group = 37.84° ± 5.05). These results do somewhat agree with previous work [24] in that the slower group shows greater levels of variability in both cases. However, it would seem ill-advised to make general statements based on the strength of these results alone as only two comparisons resulted in a significant difference.

### 4.5. CV% Relationship Testing

The lack of any statistically significant correlations between CV% and Time_TT_ suggests that there is no relationship between the level of intra-individual movement variability employed by participants and the performance outcome. The general trend, however, shows that the relationships reported are mostly negative in nature. Despite the majority of these relationships failing to reach the ‘moderate’ [37] r = ±0.400 threshold, their negative nature does suggest that a greater level of movement variability may be related to faster completion times for the time trial event. This is in direct contradiction of previous findings [24], which concluded that movement variability is not beneficial to cycling performance and, instead, agreed with previous statements that variability in motion is considered to reflect a less stable coordinative state [30], and that this may be indicative of the reduced effective force application [31].

### 4.6. Summary and Limitations

In summary, there appears to be some limited evidence of differences in the levels of intra-individual movement variability employed by different levels of cyclist during indoor cycling time trials. It should be noted that this investigation is limited to only flexion/extension couplings in the sagittal plane at the expense of movements in the other anatomical planes, but the lack of differences reported here may be somewhat explained if the results are viewed from a dynamical systems perspective.

There is growing support for the notion that intra-individual movement variability may perform a functional role in task performance [13], especially when the task requires adaptability of complex motor patterns within dynamic performance environments [14,15]. By using a cycle ergometer in a laboratory setting, it is possible that the dynamic element of the performance environment has been controlled to such a degree that there is not enough demand placed on the system to require a variable response. Removing task perturbations such as variations in road surface, weather conditions and gradient may have limited the amount of intra-individual movement variability the cyclists needed to exhibit to complete the task. As a result, this study may not give an ecologically valid representation of the functional role intra-individual movement variability can play.

Linked to this is also the inherent lack of ecological validity when using a cycle ergometer to replicate the overground cycling action. Authors have previously shown a significant difference in cycling speed and power output between laboratory and road conditions during time trial events [38,39], while others have shown that crank torque profiles are significantly different when comparing laboratory and outdoor cycling conditions [40]. As a result, future research should aim to investigate the intra-individual movement variability employed by cyclists of differing levels during outdoor cycling to further understand its role within this sport.

Finally, it must be acknowledged that the participants recruited for this investigation were all trained cyclists and, as such, relatively similar in terms of expertise. Although there was a range of competitive categories (British Cycling Race License Category 1 n = 1, Category 2 n = 2, Category 3 n = 2, Category 4 n = 5) and mean training load (self-reported as 10.85 ± 4.21 h or 156.00 ± 48.35 miles per week) among the participants, it could be argued that the relative similarity of the participants within this limited sample affects the generalisability of these findings.

It is important to note, however, that it is very difficult to recruit a true “novice” cyclist who would be capable of completing a ten-mile time trial (as required for this investigation) and even more unlikely that participants of such different experience levels would exhibit similar V^·^O_2_ max values given the amount of training required to progress through the race licence categories.

## 5. Conclusions

The results presented here suggest two significant negative linear correlations between the level of movement variability displayed by cyclists and the time taken for them to complete a cycling time trial. In addition, statistically significant differences in the level of movement variability displayed by differing levels of cyclist were seen at two time points. This suggests that there is a link between the level of intra-individual movement variability displayed by a cyclist and the time in which they were able to complete a 10-mile simulated time trial task in laboratory conditions. That this relationship is only evident at certain time points could be due to a lack of task perturbations in the laboratory setting, and therefore further research during outdoor cycling, as well as investigation of muscular recruitment patterns, is needed to understand the influence of environmental factors that are present during road cycling before the role of intra-individual movement variability can be fully understood.

## Figures and Tables

**Table 1:** Participant descriptive statistics.

	Age (Years)	Height (Metres)	Mass (Kg)	Max One Minute Power (W)	Max One Minute Power (W·Kg^−1^)	V·O_2_ max (ml·Kg^−1^·min^−1^)
Mean	31.90	1.80	72.10	365.50	5.13	73.21
Standard Deviation	10.30	0.10	9.40	69.20	0.53	12.24

**Table 2:** Mean (±standard deviation) CRPv values (°) across 10 pedal revolutions for whole-group data.

	5 min		10 min		15 min		20 min	
Pedal Phase	Hip–Knee	Knee–Ankle	Hip–Knee	Knee–Ankle	Hip–Knee	Knee–Ankle	Hip–Knee	Knee–Ankle
**Top**	3.31 (±1.35)	28.74 (±0.39)	3.70 (±0.24)	29.40 (±4.29)	3.31 (±1.53)	35.06 (±9.52)	2.70 (±0.17)	28.68 (±7.42)
**Drive**	2.36 (±0.95)	19.80 (±0.89)	2.61 (±0.08)	18.65 (±0.59)	2.38 (±0.83)	19.47 (±3.10)	2.19 (±0.20)	17.12 (±2.88)
**Bottom**	3.05 (±1.10)	16.37 (±2.14)	3.24 (±0.29)	17.80 (±6.26)	3.38 (±1.10)	15.12 (±2.87)	3.00 (±0.06)	21.19 (±5.49)
**Recovery**	4.52 (±2.71)	21.88 (±2.79)	4.44 (±1.26)	26.20 (±4.87)	4.69 (±2.45)	26.82 (±5.23)	3.94 (±2.35)	23.12 (±8.32)

**Table 3:** Mean (±standard deviation) CRPv values (°) across 10 pedal revolutions for faster-group data.

	5 min		10 min		15 min		20 min	
Pedal Phase	Hip–Knee	Knee–Ankle	Hip–Knee	Knee–Ankle	Hip–Knee	Knee–Ankle	Hip–Knee	Knee–Ankle
**Top**	3.62 (±1.78)	27.07 (±6.34)	3.05 (±0.59)	23.02 (±6.39)	3.77 (±2.79)	30.50 (±8.49)	2.07 (±0.54)	24.57 (±0.59)
**Drive**	2.77 (±1.55)	18.42 (±1.09)	2.57 (±0.02)	15.43 (±2.18)	2.82 (±1.75)	19.37 (±1.06)	2.10 (±1.00)	16.80 (±1.73)
**Bottom**	3.10 (±1.63)	18.51 (±2.91)	2.68 (±0.22)	16.65 (±7.28)	3.48 (±1.92)	16.76 (±4.72)	2.64 (±1.44)	28.00 (±9.36)
**Recovery**	3.58 (±2.11)	21.85 (±3.52)	2.41 (±0.62)	20.41 (±2.62)	3.71 (±1.59)	20.43 (±9.75)	1.82 (±0.64)	15.23 (±2.85)

**Table 4:** Mean (± standard deviation) CRPv values (°) across 10 pedal revolutions for slower-group data.

	5 min		10 min		15 min		20 min	
Pedal Phase	Hip–Knee	Knee–Ankle	Hip–Knee	Knee–Ankle	Hip–Knee	Knee–Ankle	Hip–Knee	Knee–Ankle
**Top**	2.99 (±0.92)	30.40 (±5.56)	4.23 (±0.90)	34.51 (±2.61)	3.00 (±0.80)	38.14 (±11.17)	2.97 (±0.43)	30.12 (±10.92)
**Drive**	1.95 (±0.37)	21.19 (±0.69)	2.64 (±0.12)	21.23 (±0.67)	1.99 (±0.12)	19.75 (±4.43)	2.16 (±0.23)	16.86 (±5.28)
**Bottom**	3.00 (±0.57)	14.23 (±1.37)	3.69 (±0.70)	18.72 (±5.44)	3.23 (±0.55)	14.25 (±2.02)	3.06 (±0.61)	17.19 (±2.14)
**Recovery**	5.45 (±3.31)	21.92 (±2.06)	6.06 (±2.76)	30.83 (±6.68)	5.42 (±3.21)	30.50 (±3.65)	4.79 (±3.54)	26.82 (±9.74)

**Table 5:** Correlation coefficients for CV% of CRP values against Time_TT_.

Analysis Mode	Joint Coupling	Phase	r	*p*
Full revolution	Hip–Knee		−0.375	0.285
	Knee–Ankle		−0.126	0.728
Two-Phase	Hip–Knee	Power	−0.218	0.544
		Recovery	−0.096	0.793
	Knee–Ankle	Power	−0.144	0.691
		Recovery	−0.489	0.152
Four-Phase	Hip–Knee	Top	−0.017	0.962
		Drive	0.019	0.958
		Bottom	0.59	0.073
		Recovery	−0.072	0.843
	Knee–Ankle	Top	−0.378	0.281
		Drive	0.082	0.821
		Bottom	−0.04	0.907
		Recovery	−0.505	0.136

## Data Availability

The data that support the findings of this study are available on reasonable request from the corresponding author. The data are not publicly available due to containing information that could compromise the privacy of research participants.
